# Drug Resistance in Leishmaniasis

**DOI:** 10.4103/0974-777X.62887

**Published:** 2010

**Authors:** Jaya Chakravarty, Shyam Sundar

**Affiliations:** *Department of Medicine, Institute of Medical Sciences, Banaras Hindu University, Varanasi-221 005, India*

**Keywords:** Antimony, Leishmaniasis, Resistance

## Abstract

The treatment options of leishmaniasis are limited and far from satisfactory. For more than 60 years, treatment of leishmaniasis has centered around pentavalent antimonials (Sb^v^). Widespread misuse has led to the emergence of Sb^v^ resistance in the hyperendemic areas of North Bihar. Other antileishmanials could also face the same fate, especially in the anthroponotic cycle. The HIV/ visceral leishmaniasis (VL) coinfected patients are another potential source for the emergence of drug resistance. At present no molecular markers of resistance are available and the only reliable method for monitoring resistance of isolates is the technically demanding *in vitro* amastigote-macrophage model. As the armametrium of drugs for leishmaniasis is limited, it is important that effective monitoring of drug use and response should be done to prevent the spread of resistance. Regimens of simultaneous or sequential combinations should be seriously considered to limit the emergence of resistance.

## INTRODUCTION

Leishmaniasis is a disease caused by the protozoan parasites belonging to the genus *Leishmania.* There are an estimated 12 million humans infected, with an incidence of 0.5 million cases of the visceral form of the disease and 1.5 to 2.0 million cases of the cutaneous form of the disease.[1] Ninety per cent of the annual global burden of visceral leishmaniasis (VL) cases occurs in India, Nepal, Bangladesh, and Brazil.[1,2] In India, about 100,000 cases of VL are estimated to occur annually. Of these, the state of Bihar accounts for more than 90% of cases.[2] Similarly, 90% of all cases of CL occur in Afghanistan, Brazil, Peru, Saudi Arabia, and Syria, while 90% of all cases of mucocutaneous leishmaniasis (MCL) occur in Bolivia, Brazil, and Peru.[3]

Most forms of leishmaniasis are zoonotic, human beings affected only secondarily, but two species of *Leishmania* can maintain arthroponotic, human-human cycle.[4] These species are *L. donovani,* the species responsible for VL in the Indian subcontinent and East Africa, and *L. tropica,* which is responsible for CL in the old World.

The emerging HIV/VL coinfection is locked in a vicious circle of mutual reinforcement. It has been reported from more than 35 countries, initially, most of these cases were from South Western Europe now there is increasing incidence in Africa (Ethiopia, Sudan).[5,6] The HIV/VL coinfected patients are another potential source for the emergence of drug resistance.[5,7] These patients have a high parasite burden and weak immune response. They respond slowly to treatment and have high relapse rates.[7,8] Further, reports of trans-mission of the infection via needle-sharing in HIV /VL coinfected patients in southern Europe threaten to convert an apparently zoonotic disease into the anthroponotic form.[5,9,10]

There is a regional variation in response to antileishmanial drugs and thus recommendations for treatment of VL vary in different regions. Pentavalent antimonial compounds (Sb^v^) remain the treatment of choice in Africa, South America, Bangladesh, Nepal and India (except North Bihar) at the dose of 20 mg/kg/day parenterally for 28-30 days. In the Mediterranean basin liposomal amphotericin B (L-AmB) is the treatment of choice for immunocompetent patients[11] The drug of choice for the treatment of HIV/VL coinfection is an extended course of L-AmB.[8,12]

In the recent years, new therapies have developed for VL e.g. L-AmB, oral miltefosine, and paramomycin. Although a number of drugs have now become available for the treatment of leishmaniasis each have limitation of either parenteral administration (except miltefosine), toxicity, long course of treatment, need for hospitalization and close monitoring. The treatment of cutaneous leishmaniasis may be local or systemic depending on the natural history of sores, the causative species, the possibility of mucosal dissemination, and the cosmetic and functional implications. Pentavalent antimonials are the treatment of choice where systemic treatment is indicated. Treatment of CL has improved through the introduction of topical formulations of paromomycin.[13,14] Whereas the immunomodulator immiquimod in combination with meglumine antimoniat has not shown any additional benefit.[15] Response to miltefosine is also seen in some forms of CL.[16]

At the same time as these new therapies are becoming available the standard pentavalent antimonials (Sb^v^) are being threatened by development of resistance. There is increasing awareness that drug treatment can be complicated by drug-host immune response interaction, variation in pharmacokinetics and variation in the sensitivity of *Leishmania* species to drugs.

The immune status of leishmaniasis patients has long been known to affect drug efficacy. This is of particular importance in relation to pentavalent antimonial treatment of diffuse cutaneous leishmaniasis (DCL)[17] and co infections with HIV in the visceral form,[6,18] where there is an absence of a specific T-cell mediated immune response and mutual exacerbation of infection.

The pharmacokinetic properties of an antileishmanial drug can also determine efficacy as sitamaquine an 8-minoquinoline is well distributed to the liver[19] and is being considered for treatment of VL, whereas the antifungal itraconazole (a triazole) is well distributed to the skin[20] and has been used for the treatment of CL. Significant differences were observed between patients in the elimination rate of antimonials and area under the curve analysis suggested that differences in the length of exposure to antimony could influence clinical response in CL treatment.[21]

Moreover, there are about 20 species of *Leishmania* known to be infective to humans and there is variation in intrinsic sensitivity between *Leishmania* species to several drugs.

## ANTIMONIALS

Pentavalent antimonials sodium stibogluconate and meglumine antimonate (Glucantime) remain the first line treatment for all clinical forms of leishmaniasis, despite the variable therapeutic response and the growing concern of treatment failure. One of the reasons behind the variable response could be intrinsic difference in species sensitivity to the drug, Studies using the amastigote-macrophage model, *L. donovani* and *L. brasiliensis* were found to be three- to fivefold more sensitive to sodium stibogluconate than *L. major*, *L.tropica*, and *L. Mexicana.*[22–24]

This was also observed in a controlled clinical trial in Guatemala, which compared the cure rate to antimonials in CL caused by different species; sodium stibogluconate was seen to produce a significantly higher cure rate in patients with *L. braziliensis* (96%) lesions than those with *L. mexicana* (57%).[25]

## HISTORY OF ANTIMONY RESISTANCE

Although the selection of resistant *Leishmania* has long been a part of laboratory studies, it is only in the past 20 years that acquired resistance has become a clinical threat. The first indication of drug resistance came from North Bihar, in the early 80s, of about 30% patients not responding to the prevailing regimen of Sb^v^, which was a small daily dose (10 mg/kg; 600 mg maximum) for short duration (6 to 10 day).[26] Then two 10-day courses with a 10-day interval therapy with sodium antimony gluconate were recommended by an expert committee leading to a marked improvement in the cure rates up to 99%.[27] However, in1984, it was seen that with 20 mg/kg (maximum 600 mg) for 20 days, 86% of patients were cured and cure rate with 10mg/kg was quite low.[28] In the same year, the WHO expert committee recommended that pentavalent antimony be used in doses of 20 mg/kg up to a maximum of 850 mg for 20 days, and a repetition of similar regimen for 20 days in cases of treatment failures. The WHO recommendations was evaluated a few years later by Thakur *et al*. and it was reported that only 81% of patients were cured by this regimen, although with an extension of the treatment for 40 days, 97% of patients could be cured.[29] Three years later, the same group noted a further decline in cure rate to 71% after 20 days of treatment, and recommended extended duration of treatment in nonresponders.[30] However, by early 90s, extending the therapy to 30 days could cure only 64% of patients in a hyperendemic district of Bihar.[31] In two studies carried out under strictly supervised treatment schedules, it was observed that only about one-third of the patients could be cured with the currently prevailing regimen.[32,33] The incidence of primary unresponsiveness was 52%, whereas 8% of the patients relapsed. Incidentally, only 2% of the patients from the neighboring state of (Eastern) Uttar Pradesh (UP) failed treatment.[32] There are reports of antimony resistance spreading to the Terai regions of Nepal, especially from the district adjoining the hyperendemic areas of Bihar, where up to 30% of the patients seems to be unresponsive, though in Eastern Nepal a 90% cure rate has been reported.[34] These studies confirmed that a high level of antimony resistance existed in Bihar, whereas it was still effective in surrounding areas.

There had been speculations whether Indian *Leshmania donovani* had become truly refractory to Sb^v^ or resistance occurred because of the inadequate doses being used in Bihar. In a study to determine whether acquired drug resistance was present in Bihar, *L. donovani* isolates were taken from responders and nonresponders. *In vitro* amastigote-macrophage assay showed that isolates from patients who did respond to sodium stibogluconate treatment were threefold more sensitive, with 50% effective doses (ED_50_) around 2.5 μg Sb/ml compared to isolates from patients who did not respond (ED_50_ around 7.5 μg Sb/ml).[35] The significant differences in amastigote sensitivity supported the concept of acquired resistance in Bihar.

The reasons for the emergence of resistance were the widespread misuse of the drug. Sb^v^ was freely available in India, both qualified medical practitioners and unqualified quacks used the drug and this unrestricted availability of the drug led to rampant misuse. Almost 73% patients consulted unqualified practitioners first, most of them did not use the drug appropriately.[36] It was a common practice to start with a small dose and gradually build up to the full dose over a week; it was also advocated to have drug free periods to minimize the toxicity, especially renal toxicity and physicians split the daily dose in two injections. These practices resulted in build-up of subtherapeutic blood levels and increased tolerance of parasites to Sb^v^.

Almost half of the patients, receiving pentamidine as a second-line drug, had not received adequate antimony treatment before being labeled as refractory to Sb^v^. These facts indicated large-scale misuse of antileishmanial drugs in Bihar, contributing to development of drug resistance. There were several manufacturers of Sb^v^ in India, and quality of products were inconsistent, resulting in occasional batches being substandard and toxic, this added to the problems associated with Sb^v^ therapy causing serious toxicity and deaths related to the drug.[37]

Another reason for the growing resistance to Sb^v^ in India while it still remained sensitive all over the world could be due to the fact that leishmaniasis usually has zoonotic transmission except in the Indian subcontinent and East Africa where the transmission is largely anthroponotic. In an anthroponotic cycle once Sb^v^ resistance gets established, it spreads exponentially and organisms sensitive to the drug get eliminated quickly, whereas the drug-resistant parasites continue to circulate in the community.

HIV/VL coinfected patients is another subset who respond poorly to Sb^v^, as the drug needs an intact immune system to be effective, and the response is not as good as in immunocompetent patients. Initial parasitological cure with Sb^v^ could be as low as 37%,[38] and eventually most of the initially cured patients tend to relapse. Thus, they are a potential source for emergence of drug resistance.

In CL the response is not as predictable, because there is considerable variation in sensitivity to Sb^v^ among primary isolates from untreated patients with cutaneous leishmaniasis, which correlates with patients' response to treatment.[39] Except Bihar, primary resistance is quite uncommon, but resistance develops in patients with VL, CL, and MCL who have relapsed. Chances of response to further courses of antimonials diminish once there is a relapse after the initial Sb^v^ treatment.[40] In *L. infantum* isolates taken from VL patients in France drug-sensitive strains (ED50 of<40 μg/ml) were isolated from patients who responded quickly to the meglumine treatment, whereas all the strains which were resistant under *in vitro* conditions (ED50 of>70 μg/ml) corresponded to clinical failures and *in vitro* sensitivity of strains decreased progressively in relapsing patients treated with meglumine.[41]

## MECHANISM OF RESISTANCE

The mechanism of action of antimonials are still unclear. The unique thiol metabolism of Leishmania is thought to play a pivotal role in the mechanism of action of antimonial drugs. In these parasites, the major low-molecular-mass thiol is trypanothione (T[SH]_2_).[42] Key functions of this essential metabolite include maintenance of thiol redox homeostasis, as well as defense against chemical[43] and oxidative stress.[42] Antimonial drugs are administered as pentavalent antimony [Sb^[V]^), a prodrug requiring conversion to the trivalent form [Sb(III)], before becoming biologically active. However, the site of reduction (host macrophage, amastigote, or both) and mechanism of reduction (enzymatic or nonenzymatic) remain unclear.[44,45] Sb(III) interferes directly with thiol metabolism, decreasing thiol-buffering capacity in drug-sensitive *Leishmania donovani* by inducing rapid efflux of intracellular T[SH]_2_ and GSH.[46] Sb(III) also inhibits T[SH]_2_ reductase in intact cells, resulting in the accumulation of the disulfide forms of both T[SH]_2_ (T[S]_2_) and GSH. These two mechanisms act synergistically against Leishmania parasites, leading to a lethal imbalance in thiol homeostasis.

Some studies have reported apoptosis in Sb (III)-treated amastigotes involving DNA fragmentation and externalization of phosphatidylserine on the outer surface of the plasma membrane.[47,48] However, these effects do not involve the classical caspase mediated pathway[47] and do not meet the more recent stringent definition of apoptosis.[49]

Extensive research has been done to elucidate the mechanism of resistance to antimonials, however, the exact mechanism is still not known Most of our understanding of mechanism of resistance to antimony stems from work on laboratory mutants, mostly of *Leishmania tarentolae*, in which resistance has been introduced *in vitro* by the selective pressure of heavy metals, principally arsenite and which are found to be cross resistant to Sb(III) While evaluating resistance mechanisms in the field, it should be kept in mind that *L. tarentolae* is quite different in sensitivity to antimony as compared to species that infect mammals. Further, the promastigote cell lines selected for Sb^v^ resistance may have been selected for resistance to an *m*-chlorocresol preservative which also have antileishmanial properties instead of Sb^v^ as promastigotes are not sensitive to pentavalent antimonials. Alternatively, Sb^v^ preparations could be partially reduced to Sb (III) due to prolonged storage at acidic pH or in culture media containing thiols. Some of the possible mechanisms which can lead to antimony resistance in *Leishmania* are being mentioned.

Diminished biological reduction of Sb^v^ to Sb (III) has been demonstrated in *L. donovani* amastigotes resistant to sodium stibogluconate.[50] It is not known whether this mechanism occurs in clinical isolates at present. Although recently an arsenate reductase gene *(LmACR2)* and a thiol-dependent reductase (TDRl) from *L. major* has been identified their role in drug resistance is not known.[51,52]

In prokaryotes and eukaryotes (yeast and mammalian), aquaglyceroporins (AQPs) are known to transport trivalent metalloids. Aquaglyceroporins from *L. major* (LmAQPl) have recently been demonstrated to mediate uptake of Sb(III) in *Leishmania* spp. and overexpression of aquaglycoporin 1 in drug resistant parasites is seen to render them hypersensitive to Sb(III).[53]

Increased levels of trypanothione(TSH) have been observed in some lines selected for resistance to Sb(III) or arsenite.[54] This is due to increased levels of the rate-limiting enzymes involved in the synthesis of glutathione (glutamylcysteine synthetase, GCS) and polyamines (ornithine decarboxylase, ODC) the two precursor metabolites to trypanothione.[55,56] The modulation of TSH levels by using specific inhibitors of γ-GCS or ODC could revert the resistance in mutants.[56]

The ATP-binding cassette (ABC) protein PGPA (renamed as MRPA). has been assumed to play a major role on metal resistance in *Leishmania.*[57] PGPA is a member of the multidrug-resistance protein (MRP) family, a large family of ABC transporters, several of which are implicated in drug resistance.[58] The *PGPA* gene has been shown to be frequently amplified in *Leishmania* cells that are selected for resistance to arsenite- or antimony-containing drugs.[59,60] Legare *et al*. observed that PGPA is localized in small vesicles near flagellar pocket and these are responsible for intracellular sequestration of arsenic/antimony-thiol conjugates, thereby conferring arsenite and antimonite resistance.[61] In a study on *Leishmania infantum* amastigote parasites selected for resistance to Sb(III) the expression of three genes coding for the ABC transporter MRPA (PGPA), S-adenosylhomocysteine hydrolase, and folylpolyglutamate synthase were found to be consistently increased. Transfection of the *MRPA* gene was shown to confer sodium stibogluconate resistance in intracellular parasites which could be reverted by using the glutathione biosynthesis-specific inhibitor buthionine sulfoximine.[62]

However, in an isolate from Sb^v^ refractory patients no amplified *PGPA* sequence could be detected, instead a novel 1.254-kb gene whose locus is on chromosome 9 involved in protein phosphorylation was identified.[63] Transfection experiments established that this isolated fragment confers antimony resistance to wild-type *Leishmania* species. It remains to be established whether this recently identified gene sequence can be used as a probe in the clinic to identify antimony-resistant clinical isolates on the Indian subcontinent.

Pentamidine is another antileishmanial which suffered the same fate as Sb^v^ in North Bihar. It was the first drug to be used in patients refractory to Sb^v^ and cured 99% of these patients initially however in the next two decades its efficacy dwindled to approximately 70% of patients.[64,65] Its use in VL was ultimately abandoned due to its decreased efficacy and serious toxicities. However, it has been used to good effect in treatment of both Old and New World CL and MCL. Fewer injections over short periods result in a high cure rate with minimum toxicity. In CL caused by *L. guyanensis,* 89% of cases were cured with two injections (4 mg/kg) given 48 h apart, and 80% of remaining patients were cured by a second course with minimum adverse effects.[66] In Colombian CL, four doses of 2 mg/kg of pentamidine on alternate days cured 84% patients, and four injections of 3mglkg cured 94%.[67] Its efficacy has also been demonstrated in Brazilian CLand MCL.[68–70]

The antileishmanial mechanism of action of pentamidine, are still not clearly known, however possible mechanism include inhibition of polyamine biosynthesis, DNA minor groove binding, and effect on mitochondrial inner membrane potential.[71] Pentamidine-resistant promastigote clones of *L. donovani* and *L.amazonensis* were shown to have 18- and 75-fold reduced uptakes, respectively, and increased efflux.[72] Specific transporters for pentamidine uptake have been characterized and might have a role in resistance.[71,73] Wild-type promastigotes accumulate more pentamidine in the mitochondrion in comparison to resistant cells. It is suggested that less organelle accumulation makes far more drug available for efflux.[72]

Amphotericin B a polyene antibiotic is now being used as a first line therapy in areas with Sb^v^ resistance. It has excellent cure rates (~100%) at doses of 0.75–1.00 mg/kg for 15 infusions on daily or alternate days. It has been used extensively in Bihar with uniformly good results.[74,75]

Lipid-associated amphotericin preparations are as effective as conventional amphotericin B, and have negligible adverse reactions. The dose requirement of liposomal amphotericin B varies from region to region; while in the Indian subcontinent a small dose induces high cure rates a higher dose in needed for Eastern Africa, the Mediterranean region and Brazil.[76–78] This higher efficacy of liposomal amphotericin B against *L. donovani* than *L. infantum/L. chagasi* infections is probably related more to parasite load and host immune status pathology than species sensitivity.[79]

To determine the mechanism of resistance, a resistant clone of *L. donovani* promastigotes was selected through a stepwise increase in amphotericin B concentration in culture. Resistant promastigotes showed a significant change in plasma membrane sterol profile by gas chromatography-mass spectrometry, ergosterol being replaced by a precursor, cholesta-5, 7, 24-trien-3β-ol.[80] This probably results from a defect in C-24 transmethylation due to loss of function of S-adenosyl-L-methionine-C24-Δ-sterolmethyltransferase (SCMT). In *L. donovani* promastigotes two transcripts of the enzyme have now been characterized, one of which was absent in the amphotericin B-resistant clone, the other overexpressed but without a splice leader sequence which would prevent translation.[81] These studies have been performed with promastigotes and their importance in the intracellular amastigote is not known.

Clinical resistance to amphotericin B is rare. Nevertheless, with the increasing use of amphotericin B, especially in lipid formulations which have longer half life, the possibility of resistance cannot be ignored. There are two small inconclusive studies on the emergence of amphotericin B resistance in *L. infantum*/HIV-infected cases in France. One study failed to find a change in sensitivity in promastigotes derived from isolates taken before and after the treatment of one patient.[82] In contrast, a decrease in sensitivity was observed in isolates taken over several relapses from another patient.[83]

Miltefosine, an alkyl phospholipid is the first oral agent approved for the treatment of leishmaniasis. At the recommended doses (100mg daily for patients weighing ≥25 kg and 50mg daily for those weighing ≤25 kg for 4 weeks) cure rates were 94% for VL.[84] It has a long-terminal half-life, which ranges between 150 and 200 h. About four half-lives (25–33 days) are required to reach more than 90% clearance of the plateau levels (at steady-state). Thus, subtherapeutic levels may remain for some weeks after a standard course of treatment. This characteristic might encourage the emergence of resistance.[85]

*In vitro* studies shows variation in the sensitivities of both promastigote and amastigote stages of *L. donovani, L. major, L. tropica, L. aethiopica, L. mexicana*, and *L. panamensis* to miltefosine.[86] In all assays *L.donovani* was the most sensitive species and *L. major* was the least sensitive species. Studies on clinical isolates using a murine macrophage-amastigote model have confirmed the high sensitivity of *L. donovani* from both Sb-sensitive and Sb resistant patients from Nepal and lack of sensitivity of *L. braziliensis* and *L. guyanensis* isolates from patients in Peru.[87] This variability in sensitivity reflects differences in intrinsic susceptibility however it could have an important impact on clinical outcome. The greatest clinical significance is seen in Central and South America where distribution of *L. mexicana, L. amazonensis, L. panamensis, L. braziliensis* overlap. The clinical relevance of this finding was observed for CL by Soto *et al*. in Colombia, where *L. panamensis* is common, the cure rate was 91%, whereas in Guatemala, where *L. braziliensis* and *L. mexicana* are common, the cure rate was 53%.[88]

Although the exact mechanism of action of miltefosine is not clear it is known to induce apoptosis-like death in *L. donovani* based on observed phenomena such as cell shrinkage, nuclear DNA condensation, DNA fragmentation into oligonucleosome-sized fragments and phosphatidylserine exposure.[89,90]

In experimental *L. donovani* strains resistant to miltefosine, the mechanism of resistance was found to be due to a >95% reduced accumulation of ^14^C-labeled miltefosine. A defect in the internalization step must have occurred in the resistant line as binding to the parasite plasma membrane, efflux of preloaded drug and metabolism were similar in sensitive and resistant parasites.[91] A novel plasma membrane P-type transporter (LdMT gene) from the aminophospholipid translocase subfamily has been observed to be responsible for the uptake of both miltefosine and glycerophospholipids into *L. donovani* promastigotes. Two alleles with single distinct point mutations on this transporter were shown to be responsible for the reduced uptake.[92] The localization of LdMT and thus its activity depends on the presence of a specific beta subunit, LdRos3 which belongs to the CDC50/Lem3 protein family. Both proteins are mutually dependent for their function and their localization at the plasma membrane.[93] However, whether the inactivation of LdMT or LdRos3 produce resistant parasites in *in vivo* situations is not known. Another mechanism for resistance could be an increase in drug efflux, mediated by the overexpression of the ABC transporter P-glycoprotein, Previously it had been shown that multidrug-resistant *L.tropica* lines that over express a P-glycoprotein are less sensitive to miltefosine.[94] In contrast, P-glycoprotein overexpression was not observed in the 40 μM-miltefosine-resistant promastigotes.[95]

Paromomycin, an aminoglycoside-aminocyclitol antibiotic, has been used for the treatment of VL in a parenteral formulation and CL in both topical and parenteral formulations. In the phase III trial of Paromomycin in the Indian subcontinent, it was shown to be noninferior to amphotericin B and was approved by the Indian government in August 2006 for the treatment of patients with visceral leishmaniasis.[96] Topical preparations of paromomycin, a soft paraffin-based ointment containing 15% of paromomycin and 12% methyl-benzethonium chloride (MBCL), are effective against both Old World as well as New World CL.[97,98] Variation in sensitivity has been seen in both experimental models and clinical cases of CL, as lesions caused by *L. major* treated with paromomycin ointment resolved faster and more completely than lesions caused by *L. amazonensis* and *L. panamensis.*[99]

A more indepth *in vitro* analysis on the sensitivity of amastigotes in a murine macrophage model showed that *L. major* and *L. tropica* were more sensitive than *L. braziliensis* and *L. mexicana* isolates and *L. donovani* showed intermediate sensitivity.[100] Clinical resistance with this drug in VL is not known as it has not been used extensively. However, following a 60-day parenteral course for treatment of CL in two *L. aethiopica* cases, isolates taken from relapsed patients were three- to fivefold less sensitive to the drug after treatment than isolates taken before treatment in an amastigote-macrophage assay.[101]

The mechanisms of action of paromomycin in *Leishmania* spp. is exactly not known however mitochondrial ribosomes and induction of respiratory dysfunction and mitochondrial membrane depolarization have been implicated.[102,103] In studies on selected populations of promastigotes, resistance was related to decreased drug uptake in *L. donovani.*[104] In a recent study, the mitochondrial membrane potential was significantly decreased after 72 hours of exposure to paromomycin indicating that this organelle might be the ultimate target of the drug. Both cytoplasmic and mitochondrial protein synthesis were inhibited, however, the drug induced reduction in membrane potential and inhibition of protein synthesis were less pronounced in the resistant strain as compared to the wild-type. A line selected for resistance to the drug showed reduced paromomycin accumulation associated with a significant reduction in the initial binding to the cell surface.[105]

Sitamaquine, a 4-methyl-6-methoxy-8-aminoquinoline has limited clinical use and no reported resistance. Relatively poor efficacy compounded with nephrotoxicity suggests that this drug cannot be used as monotherapy in VL.[106]

Azole-like ketaconazole and triazoles, intraconazole, and fluconazole have antileishmanial effects.[107] One placebo-controlled trial on the treatment of CL showed that *L. mexicana* infections (89%) were more responsive than *L. braziliensis* infections (30%) to ketoconazole indicating an intrinsic differences in sensitivity of *Leishmania* species to azoles.[108] These drugs has limited clinical use and clinical resistance is not known.

## STRATEGIES TO COMBAT DRUG RESISTANCE

### Monitoring therapy

In a study to detect the factors leading to antimony resistance in Indian VL it was observed that only 26% were treated according to the WHO guidelines, 42% did not take the drug regularly and 36% stopped the drug on their own initiative.[36] Similar concerns were raised for miltefosine when in a preliminary data from a phase IV trial in India involving domiciliary treatment with miltefosine and weekly supervision showed doubling of the relapse rate.[109] These findings suggests that monitoring therapy is imperative to prevent development of resistance. The directly observed treatment strategy (DOTS) for tuberculosis has been a big success and either a parallel or integrated with DOTS system could be evolved for leishmaniasis. This will lead to better compliance, completion of the treatment course and ultimately prevent resistance.

### Free distribution of drugs

The high cost of the antileishmanial drugs coupled with easy, over the counter availability often leads to under dosing and incomplete treatment. This has been the major factor for antimony resistance and could lead to resistance to other drugs as well especially the novel oral agent miltefosine. Considering that majority of the population cannot afford to purchase and complete a full course of treatment it is recommended that antileishmanials should be made available free of cost to be distributed through public and/ or private health care providers like antitubercular and antiretroviral drugs, and antileishmanial drugs should be withdrawn from the open market.

### Combination therapy

The growing resistance of the parasite to antileishmanial drugs suggests that the currently used monotherapy needs to be reviewed. Multidrug combination therapy has been used successfully in tuberculosis, leprosy and malaria. The rationale behind combination therapy are increased activity through use of compounds with synergistic or additive activity, preventing the emergence of drug resistance, lower dose requirement thereby reducing chances of toxic side effects and cost, and increased spectrum of activity.

Recently, a study showed that an single infusion of Liposomal Amphotericin B (at a dose 3.75 mg/kg - 5mg/kg) followed by a brief (7, 10 or 14 days) self-administered course of miltefosine had excellent cure rates making it a feasible option for Indian *kala-azar*.[110] The preferential pricing agreement with WHO has reduced the price of Liposomal Amphotericin B (AmBisome®) for endemic regions to $20 per 50-mg vial and this further opens the prospect of combining of Liposomal Amphotericin B in various combination regimens.[111] Further studies to identify combination therapy with drugs like lipid formulations of amphotericin B, miltefosine and paramomycin are underway with 8-11 days duration of therapy. If successful, this would be a groundbreaking find providing affordable treatment with much improved compliance and prevent the emergence of resistance. The pipeline for the antileishmanial drug is empty, it is imperative that we try and protect and prolong the effective life of the existing drugs.

### Monitoring drug resistance

Ideally, parasite resistance should be monitored, rather than relapses or unresponsiveness. It will also permit the identification of key intracellular targets and parasite defense mechanisms, which can then be exploited to rationally develop analogues of existing drugs that would not affected by the most common defenses. Analysis of genetic markers that determine high antileishmanial resistance, performed systematically for every parasite isolate that shows low antileishmanial sensitivity would facilitate the tracking of the level of resistance in affected populations. At present there are no molecular markers of resistance available for the currently used antileishmanial drugs and the only reliable method for monitoring resistance of isolates is the technically demanding *in vitro* amastigote-macrophage model. Development of drug resistance markers and tools easy to use in the field should be encouraged.

### Management of HIV/VL Co-infection

Another potential source for the emergence of drug resistance are the HIV/VL coinfected patients. These patients have high parasite burden, a weak immune response, respond poorly to treatment and have a high relapse rate. Therefore they are the ideal candidates to harbor drug resistant parasites. With the growing burden of HIV in India, HIV/VL coinfection could become a major problem. Experience from Southern Europe shows that initial response to Sb^v^ and conventional amphotericin B is low (~40-65%) in severely immuncompromised persons and severe adverse events are frequent. Initiation of HAART dramatically decreases the incidence of VL coinfection. Therefore; HAART in combination with antileishmanials should be advocated strictly in these patients.

## CONCLUSION

Inventory of antileishmanial drugs is very small, and emergence of drug resistance is further complicating the control of leishmaniasis. A better understanding of mechanism of action of the drugs and unraveling the puzzle of drug resistance mechanisms, with easy to use markers of resistance may pave the way for more rational use of drugs. Combination chemotherapy is rapidly emerging as the norm for treating several infective disorders like malaria, tuberculosis, HIV etc., and its application is strongly advocated for VL. Directly observed therapy given free, in treatment centers manned by trained personnel, will go a long way in controlling the disease as well as drug resistance.

## References

[1] Desjeux P (1992). Human leishmaniases: epidemiology and public health aspects. World Health Stat Q.

[2] Bora D (1999). Epidemiology of visceral leishmaniasis in India. Natl Med J India.

[3] Richens J (2004). Genital manifestations of tropical diseases. Sex Transm Infect.

[4] Magill AJ (1995). Epidemiology of the leishmaniases. Dermatol Clin.

[5] Alvar J, Cañavate C, Gutiérrez-Solar B, Jiménez M, Laguna F, López-Vélez R (1997). Leishmania and human immunodeficiency virus coinfection: the first 10 years. Clin Microbiol Rev.

[6] Desjeux P, Alvar J (2003). LeishmaniaIHIV co-infections: epidemiology in Europe. Ann Trop Med Parasitol.

[7] Laguna F, Videla S, Jiménez-Mejías ME, Sirera G, Torre-Cisneros J, Ribera E (2003). Amphotericin B lipid complex versus meglumine antimoniate in the treatment of visceral leishmaniasis in patients infected with HIV: a randomized pilot study. J Antimicrob Chemother.

[8] Russo R, Nigro LC, Minniti S, Montineri A, Gradoni L, Caldeira L (1996). Visceral leishmaniasis in HIV infected patients: treatment with high dose liposomal amphotericin B (AmBisome). J Infect.

[9] Alvar J, Gutiérrez-Solar B, Pachón I, Calbacho E, Ramírez M, Vallés R (1996). AIDS and *Leishmania infantum*. New approaches for a new epidemiological problem. Clin Dermatol.

[10] Molina R, Gradoni L, Alvar J (2003). HIV and the transmission of Leishmania. Ann Trop Med Parasitol.

[11] Cascio A, Colomba C (2003). Childhood Mediterranean visceral leishmaniasis. Infez Med.

[12] Davidson RN, Di Martino L, Gradoni L, Giacchino R, Russo R, Gaeta GB (1994). Liposomal amphotericin B (AmBisome) in Mediterranean visceral leishmaniasis: a multicentre trial. Q J Med.

[13] Asilian A, Jalayer T, Nilforooshzadeh M, Ghassemi RL, Peto R, Wayling S (2003). Treatment of cutaneous leishmaniasis with aminosidine (paromomycin) ointment: double-blind, randomized trial in the Islamic Republic of Iran. Bull World Health Organ.

[14] el-On J, Halevy S, Grunwald MH, Weinrauch L (1992). Topical treatment of Old World cutaneous leishmaniasis caused by *Leishmania major*: a double-blind control study. J Am Acad Dermatol.

[15] Firooz A, Khamesipour A, Ghoorchi MH, Nassiri-Kashani M, Eskanolari SE, Khatami A (2006). Imiquimod in combination with meglumine antimoniate for cutaneous leishmaniasis. Arch Dermatol.

[16] Soto J, Arana BA, Toledo J, Rizzo N, Vega JC, Diaz A (2004). Miltefosine for new world cutaneous leishmaniasis. Clin Infect Dis.

[17] Ercoli N (1966). Drug responsiveness in experimental cutaneous leishmaniosis. Exp Parasitol.

[18] Berhe N, Wolday D, Hailu A, Abraham Y, Ali A, Gebre-Michael T (1999). HIV viral load and response to antileishmanial chemotherapy in co-infected patients. AIDS.

[19] Carson PE, Peters W, Richards WH (1984). 8-aminoquinolines. Antimalarial drugs II.

[20] Leyden J (1998). Pharmacokinetics and pharmacology of terbinafine and itraconazole. J Am Acad Dermatol.

[21] Al-Jaser M, El-Yazigi A, Croft SL (1995). Pharmacokinetics of antimony in patients treated with sodium stibogluconate for cutaneous leishmaniasis. Pharm Res.

[22] Allen S, Neal RA, Hart DT (1998). The *in vitro* susceptibility of macrophages infected with amastigotes of *Leishmania* spp.to pentavalent antimonial drugs and other compounds with special relevance to cutaneous isolates. Leishmaniasis.

[23] Berman JD (1981). Activity of imidazoles against *Leishmania tropica* in human macrophage cultures. Am J Trop Med Hyg.

[24] Neal RA, Allen S, McCoy N, Olliaro P, Croft SL (1995). The sensitivity of *Leishmania* species to aminosidine. J Antimicrob Chemother.

[25] Navin TR, Arana BA, Arana FE, Berman JD, Chajon JF (1992). Placebo-controlled clinical trial of sodium stibogluconate (Pentostam) versus ketoconazole for treating cutaneous leishmaniasis in Guatemala. J Infect Dis.

[26] Peters W (1981). The treatment of *kala-azar*. New approach to an old problem. Indian J Med Res.

[27] Aikat BK, Sahaya S, Pathania AG, Bhattacharya PK, Desai N, Prasad LS (1979). Clinical profile of cases of *kala-azar* in Bihar. Indian J Med Res.

[28] Thakur CP, Kumar M, Singh SK, Sharma D, Prasad US, Singh RS (1984). Comparison of regimens of treatment with sodium stibogluconate in *kala-azar*. Br Med J (Clin Res Ed).

[29] Thakur CP, Kumar M, Kumar P, Mishra BN, Pandey AK (1988). Rationalisation of regimens of treatment of *kala-azar* with sodium stibogluconate in India: a randomised study. Br Med J (Clin Res Ed).

[30] Thakur CP, Kumar M, Pandey AK (1991). Evaluation of efficacy of longer durations of therapy of fresh cases of *kala-azar* with sodium stibogluconate. Indian J Med Res.

[31] Jha TK, Singh NK, Jha SN (1992). Therapeutic use of sodium stibogluconate in *kala-azar* from some hyperendemic districts of N.Bihar, India (Abstract). J Assoc Physicians India.

[32] Sundar S, More DK, Singh MK, Singh VP, Sharma S, Makharia A (2000). Failure of pentavalent antimony in visceral leishmaniasis in India: report from the center of the Indian epidemic. Clin Infect Dis.

[33] Sundar S, Singh VP, Sharma S, Makharia MK, Murray HW (1997). Response to interferon-ά plus pentavalent antimony in Indian visceral leishmaniasis. J Infect Dis.

[34] Rijal S, Chappuis F, Singh R, Bovier PA, Acharya P, Karki BM (2003). Treatment of visceral leishmaniasis in south-eastern Nepal: decreasing efficacy of sodium stibogluconate and need for a policy to limit further decline. Trans R Soc Trop Med Hyg.

[35] Lira R, Sundar S, Makharia A, Kenney R, Gam A, Saraiva E, Sacks D (1999). Evidence that the high incidence of treatment failures in Indian *kala-azar* is due to the emergence of antimony-resistant strains of Leishmania donovani. J Infect Dis.

[36] Sundar S, Thakur BB, Tandon AK, Agrawal NR, Mishra CP, Mahapatra TM (1994). Clinico-epidemiological study of drug resistance in Indian *kala-azar*. BMJ.

[37] Sundar S, Sinha PR, Agrawal NK, Srivastava R, Rainey PM, Berman JD (1998). A cluster of cases of severe cardiotoxicity among *kala-azar* patients treated with a high-osmolarity lot of sodium antimony gluconate. Am J Trop Med Hyg.

[38] Laguna F, Videla S, Jiménez-Mejías ME, Sirera G, Torre-Cisneros J, Ribera E (2003). Amphotericin B lipid complex versus meglumine antimoniate in the treatment of visceral leishmaniasis in patients infected with HIV: a randomized pilot study. J Antimicrob Chemother.

[39] Berman JD, Chulay JD, Hendricks LD, Oster CN (1982). Susceptibility of clinically sensitive and resistant Leishmania to pentavalent antimony *in vitro*. Am J Trop Med Hyg.

[40] Bryceson AD, Chulay JD, Ho M, Mugambii M, Were JB, Muigai R (1985). Visceral leishmaniasis unresponsive to antimonial drugs. 1. Clinical and immunological studies. Trans R Soc Trop Med Hyg.

[41] Faraut-Gambarelli F, Piarroux R, Deniau M, Giusiano B, Marty P, Michel G (1997). *In vitro* and in vivo resistance of *Leishmania infantum* to meglumine antimoniate: a study of 37 strains collected from patients with visceral leishmaniasis. Antimicrob Agents Chemother.

[42] Fairlamb AH, Cerami A (1992). Metabolism and functions of trypanothione in the Kinetoplastida. Annu Rev Microbiol.

[43] Vickers TJ, Greig N, Fairlamb AH (2004). A trypanothione-dependent glyoxalase I with a prokaryotic ancestry in Leishmania major. Proc Natl Acad Sci USA.

[44] Frezard F, Demicheli C, Ferreira CS, Costa MA (2001). Glutathione-induced conversion of pentavalent antimony to trivalent antimony in meglumine antimoniate. Antimicrob Agents Chemother.

[45] Shaked-Mishan P, Ulrich N, Ephros M, Zilberstein D (2001). Novel intracellular Sb-V reducing activity correlates with antimony susceptibility in Leishmania donovani. J Biol Chem.

[46] Wyllie S, Cunningham ML, Fairlamb AH (2004). Dual action of antimonial drugs on thiol redox metabolism in the human pathogen Leishmania donovani. J Biol Chem.

[47] Sereno D, Holzmuller P, Mangot I, Cuny G, Ouaissi A, Lemesre JL (2001). Antimonial-mediated DNA fragmentation in *Leishmania infantum* amastigotes. Antimicrob Agents Chemother.

[48] Sudhandiran G, Shaha C (2003). Antimonial-induced increase in intracellular Ca^2+^ through non-selective cation channels in the host and the parasite is responsible for apoptosis of intracellular *Leishmania donovani* amastigotes. J Biol Chem.

[49] Jiang X, Wang X (2004). Cytochrome C-mediated apoptosis. Annu Rev Biochem.

[50] Shaked-Mishan P, Ulrich N, Ephros M, Zilberstein D (2001). Novel Intracellular SbV reducing activity correlates with antimony susceptibility in *Leishmania donovani*. J Biol Chem.

[51] Zhou Y, Messier N, Ouellette M, Rosen BP, Mukhopadhyay R (2004). *Leishmania major* LmACR2 is a pentavalent antimony reductase that confers sensitivity to the drug Pentostam. J BioI Chern.

[52] Denton H, McGregor JC, Coombs GH (2004). Reduction of anti-leishmanial pentavalent antimonial drugs by a parasite-specific thiol-dependent reductase, TDRI. Biochem J.

[53] Gourbal B, Sonuc N, Bhattacharjee H, Legare D, Sundar S, Ouellette M (2004). Drug uptake and modulation of drug resistance in Leishmania by an aquaglyceroporin. J Biol Chem.

[54] Mukhopadhyay R, Dey S, Xu N, Gage D, Lightbody J, Ouellette M (1996). Trypanothione overproduction and resistance to antimonials and arsenicals in *Leishmania*. Proc Natl Acad Sci USA.

[55] Grondin K, Haimeur A, Mukhopadhyay R, Rosen BP, Ouellette M (1997). Co-amplification of the gamma-glutamylcysteine synthetase gene gsh1 and of the ABC transporter gene pgpA in arsenite-resistant Leishmania tarentolae. EMBO J.

[56] Haimeur A, Guimond C, Pilote S, Mukhopadhyay R, Rosen BP, Poulin R (1999). Elevated levels of polyamines and trypanothione resulting from overexpression of the ornithine decarboxylase gene in arsenite-resistant Leishmania. Mol Microbiol.

[57] Ouellette M, Legare D, Haimeur A, Grondin K, Roy G, Brochu C, Papadopoulou B (1998). ABC transporters in *Leishmania* and their role in drug resistance. Drug Resist Updat.

[58] Higgins CF (1992). ABC transporters: from microorganisms to man. Annu Rev Cell Bioi.

[59] Callahan HL, Beverley SM (1991). Heavy metal resistance: a new role for P-glycoproteins in Leishmania. J Biol Chem.

[60] Ouellette M, Borst P (1991). Drug resistance and P-glycoprotein gene amplification in the protozoan parasite Leishmania. Res Microbiol.

[61] Legare D, Richard D, Mukhopadhyay R, Stierhof YD, Rosen BP, Haimeur A (2001). The *Leishmania* ATP-binding cassette protein PGPA is an intracellular metal-thiol transporter ATPase. J Biol Chem.

[62] El Fadili K, Messier N, Leprohon P, Roy G, Guimond C, Trudel N (2005). Role of the ABC transporter MRPA (PGPA) in antimony resistance in *Leishmania infantum* axenic and intracellular amastigotes. Antimicrob Agents Chemother.

[63] Singh N, Singh RT, Sundar S (2003). Novel mechanism of drug resistance in kala azar field isolates. J Infect Dis.

[64] Jha SN, Singh NK, Jha TK (1991). Changing response to diamidine compounds in cases of *kala-azar* unresponsive to antimonial. J Assoc Physicians India.

[65] Jha TK (1983). Evaluation of diamidine compound (pentamidine isethionate) in the treatment resistant cases of *kala-azar* occurring in North Bihar, India. Trans R Soc Trop Med Hyg.

[66] Nacher M, Carme B, Sainte Marie D, Couppié P, Clyti E (2001). Influence of clinical presentation on the efficacy of a short course of pentamidine in the treatment of cutaneous leishmaniasis in French Guiana. Ann Trop Med Parasitol.

[67] Soto 1, Buffet P, Grogl M, Berman 1 (1994). Successful treatment of Colombian cutaneous leishmaniasis with four injections of pentamidine. Am 1 Trop Med Hyg.

[68] Correia D, Macêdo VO, Carvalho EM, Barral A, Magalhães AV, de Abreu MV (1996). Comparative study of meglumine antimoniate, pentamidine isethionate and amino-sidine sulfate in the treatment of primary skin lesions caused by Leishmania (Viannia) braziliensis. Rev Soc Bras Med Trop.

[69] de Paula CD, Sampaio JH, Cardoso DR, Sampaio RN (2003). A comparative study between the efficacy of pentamidine isothionate given in three doses for one week and N-methil-glucamine in a dose of 20 mg Sb V/day for 20 days to treat cutaneous leishmaniasis. Rev Soc Bras Med Trop.

[70] Amato V, Amato J, Nicodemo A, Uip O, Amato-Neto V, Duarte M (1998). Treatment of mucocutaneous leishmaniasis with pentamidine isothionate. Ann Dermatol Venereol.

[71] Bray PG, Barrett MP, Ward SA, de Koning HP (2003). Pentamidine uptake and resistance in pathogenic protozoa: past, present and future. Trends Parasitol.

[72] Basselin M, Denise H, Coombs GH, Barrett MP (2002). Resistance to pentamidine in *Leishmania mexicana* involves exclusion of the drug from the mitochondrion. Antimicrob Agents Chemother.

[73] Coelho AC, Beverley SM, Cotrim PC (2003). Functional genetic identification of PRP1, an ABC transporter superfamily member conferring pentamidine resistance in *Leishmania major*. Mol Biochem Parasitol.

[74] Thakur CP, Singh RK, Hassan SM, Kumar R, Narain S, Kumar A (1999). Amphotericin B deoxycholate treatment of visceral leishmaniasis with newer modes of administration and precautions: a study of 938 cases. Trans R Soc Trop Med Hyg.

[75] Mishra M, Biswas UK, lha DN, Khan AB (1992). Amphotericin versus pentamidine in antimony-unresponsive *kala-azar*. Lancet.

[76] Sundar S, Agrawal NK, Sinha PR, Horwith GS, Murray HW (1997). Short-course, low-dose amphotericin B lipid complex therapy for visceral leishmaniasis unresponsive to antimony. Ann Intern Med.

[77] Sundar S, Jha TK, Thakur CP, Mishra M, Singh VR, Buffels R (2002). Low-dose liposomal amphotericin B in refractory Indian visceral leishmaniasis: a multicenter study. Am J Trop Med Hyg.

[78] Berman JD (1999). DS Food and Drug Administration approval of AmBisome (liposomal amphotericin B) for treatment of visceral leishmaniasis. Clin Infect Dis.

[79] Berman JD, Badaro R, Thakur CP, Wasunna KM, Behbehani K, Davidson R (1998). Efficacy and safety of liposomal amphotericin B (AmBisome) for visceral leishmaniasis in endemic developing countries. Bull World Health Organ.

[80] Mbongo N, Loiseau PM, Billion MA, Robert-Gero M (1998). Mechanism of amphotericin B resistance in *Leishmania donovani* promastigotes. Antimicrob Agents Chemother.

[81] Pourshafie M, Morand S, Virion A, Rakotomanga M, Dupuy C, Loiseau PM (2004). Cloning of S-adenosyl-L-methionine:C-24-Δ-sterolmethyltransferase (ERG6) from *Leishmania donovani* and characterization of mRNAs in wild-type and amphotericin B-resistant promastigotes. Antimicrob Agents Chemother.

[82] Durand R, Paul M, Pratlong F, Rivollet D, Dubreuil-Lemaire ML, Houin R (1998). *Leishmania infantum*: lack of parasite resistance to amphotericin B in a clinically resistant visceral leishmaniasis. Antimicrob Agents Chemother.

[83] Di Giorgio C, Faraut-Gambarelli F, Imbert A, Minodier P, Gasquet P, Dumon H (1999). Flow cytometric assessment of amphotericin B susceptibility in *Leishmania infantum* isolates from patients with visceral leishmaniasis. J Antimicrob Chemother.

[84] Sundar S, Makharia A, More DK, Agrawal G, Voss A, Fischer C (2000). Short-course miltefosine treatment for visceral leishmaniasis. Clin Infect Dis.

[85] Bryceson A (2001). A policy for leishmaniasis with respect to the prevention and control of drug resistance. Trop Med Int Health.

[86] Escobar P, Matu S, Marques C, Croft SL (2002). Sensitivities of *Leishmania* species to hexadecylphosphocholine (miltefosine), ET-18-OCH(3) (edelfosine) and amphotericin B. Acta Trop.

[87] Yardley V, Croft SL, De Donker S, Dujardin JC, Siddhartha K, Miranda C (2005). The sensitivity of clinical isolates of *Leishmania* from Peru and Nepal to miltefosine. Am J Trop Med Hyg.

[88] Soto J, Arana BA, Toledo J, Rizzo N, Vega JC, Diaz A (2004). Miltefosine for new world cutaneous leishmaniasis. Clin Infect Dis.

[89] Verma NK, Dey CS (2004). Possible mechanism of miltefosinemediated death of *Leishmania donovani*. Antimicrob Agents Chemother.

[90] Paris C, Loiseau PM, Bories C, Breard J (2004). Miltefosine induces apoptosis-like death in *Leishmania donovani* promastigotes. Antimicrob Agents Chemother.

[91] P'erez-Victoria FJ, Castanys S, Gamarro F (2003). Resistance to miltefosine in *Leishmania donovani* involves a defective inward translocation of the drug. Antimicrob Agents Chemother.

[92] Perez-Victoria FJ, Gamarro F, Ouellette M, Castanys S (2003). Functional cloning of the miltefosine transporter. A novel P-type phospholipid translocase from *Leishmania* involved in drug resistance. J Biol Chem.

[93] Pérez-Victoria FJ, Sanchez-Canete MP, Castanys S, Gamarro F (2006). Phospholipid translocation and miltefosine potency require both *L.donovani* miltefosine transporter and the new protein LdRos3 in *Leishmania* parasites. J Biol Chem.

[94] Perez-Victoria FJ, Di Pietro A, Barron D, Ravelo AG, Castanys S, Gamarro F (2002). Multidrug resistance phenotype mediated by the P-glycoprotein-like transporter in *Leishmania*: a search for reversal agents. Curr Drug Targets.

[95] Seifert K, Matu S, Perez Victoria FJ, Castamys S, Gamarro F, Croft SL (2003). Characterization of *Leishmania donovani* promastigotes resistant to hexadecylphophocholine (miltefosine). Int J Antimicrob Agents.

[96] Sundar S, Jha TK, Thakur CP, Sinha PK, Bhattacharya SK (2007). Injectable paromomycin for visceral leishmaniasis in India. N Engl J Med.

[97] el-On J, Halevy S, Grunwald MH, Weinrauch L (1992). Topical treatment of Old World cutaneous leishmaniasis caused by *Leishmania major*: a double-blind control study. J Am Acad Dermatol.

[98] Krause G, Kroeger A (1994). Topical treatment of American cutaneous leishmaniasis with paramomycin and methylbenzethonium chloride: a clinical study under field conditions in Ecuador. Trans R Soc Trop Med Hyg.

[99] el-On J, Hamburger AD (1987). Topical treatment of New and OldWorld cutaneous leishmaniasis in experimental animals. Trans R Soc Trop Med Hyg.

[100] Neal RA, Allen S, McCoy N, Olliaro P, Croft SL (1995). The sensitivity of *Leishmania* species to aminosidine. J Antimicrob Chemother.

[101] Teklemariam S, Hiwot AG, Frommel D, Miko TL, Ganlov G, Bryceson A (1994). Aminosidine and its combination with sodium stibogluconate in the treatment of diffuse cutaneous leishmaniasis caused byLeishmania aethiopica. Trans R Soc Trop Med Hyg.

[102] Maarouf M, de Kouchkovsky Y, Brown S, Petit PX, Robert-Gero M (1997). In vivo interference of paromomycin with mitochondrial activity of Leishmania. Exp Cell Res.

[103] Maarouf M, Lawrence F, Croft SL, Robert-Gero M (1995). Ribosomes of *Leishmania* are a target for the aminoglycosides. Parasitol Res.

[104] Maarouf M, Adeline MT, Solignac M, Vautrin D, Robert-Gero M (1998). Development and characterization of paromomycin-resistant Leishmania donovani promastigotes. Parasite.

[105] Jhingram A, Chawla B, Saxena S, Barrett MP, Madhubala R (2009). Paromomycin: uptake and resistance in Leishmania donovani. Mol Biochem Parasitol.

[106] Dietze R, Carvalho SF, Valli LC, Berman J, Brewer T, Milhous W (2001). Phase 2 trial of WR6026 an orally administered 8-aminoquinoline, in the treatment of visceral leishmaniasis caused by *Leishmania chagasi*. Am J Trop Med Hyg.

[107] Croft SL, Yardley V (2002). Chemotherapy of leishmaniasis. CUIT Pharm Des.

[108] Navin TR, Arana BA, Arana FE, Berman JD, Chajon JF (1992). Placebo-controlled clinical trial of sodium stibogluconate (Pentostam) versus ketoconazole for treating cutaneous leishmaniasis in Guatemala. J Infect Dis.

[109] Sundar S, Murray HW (2005). Availability of miltefosine for the treatment of *kala-azar* in India. Bull World Health Organ.

[110] Sundar S, Rai M, Chakravarty J, Agarwal D, Agrawal N, Vaillant M (2008). New treatment approach in indian visceral leishmaniasis: single-dose liposomal amphotericin b followed by short-course oral miltefosine. Clin Infect Dis.

[111] Olliaro P, Sundar S (2009). Anthropometrically derived dosing and drug costing calculations for treating visceral leishmaniasis in Bihar, India. Trop Med Int Health.

